# One Year of Low-Titer Whole Blood Resuscitation at an Academic Level 1 Trauma Center

**DOI:** 10.7759/cureus.100699

**Published:** 2026-01-03

**Authors:** David A Leon, Christopher M Wend, Zakk Arciaga, Jace Bradshaw, Steve M Frank, Kent A Stevens, Elizabeth P Crowe, Matthew J Levy

**Affiliations:** 1 Department of Anesthesiology and Pain Medicine, University of California Davis School of Medicine, Sacramento, USA; 2 Department of Emergency Medicine, University of California Davis School of Medicine, Sacramento, USA; 3 Department of Emergency Medicine, Johns Hopkins University School of Medicine, Baltimore, USA; 4 Department of Anesthesiology and Critical Care Medicine, Johns Hopkins University School of Medicine, Baltimore, USA; 5 Department of Surgery, Division of Acute Care Surgery, Johns Hopkins University School of Medicine, Baltimore, USA; 6 Department of Pathology, Johns Hopkins University School of Medicine, Baltimore, USA; 7 Office of the Medical Director, Howard County Department of Fire and Rescue Services, Mariottsville, USA

**Keywords:** acute care surgery and trauma, balanced resuscitation, blood transfusion safety, blood transfusion serviceswhole blood, emergency medicine and trauma, massive blood transfusion

## Abstract

Objective

Whole blood has shown significant promise in the military setting for resuscitation of trauma patients at risk of massive hemorrhage. Whole blood resuscitation has been increasingly applied to civilian trauma, where hemorrhagic shock is one of the most preventable causes of death. This study describes the natural experience post-implementation of a low-titer O-positive whole blood (LTOWB) protocol for adult trauma patients who presented to our academic level 1 urban trauma center.

Methodology

This was a single-center descriptive analysis of adult patients who received LTOWB over 12 months, from program start in December 2022 to December 2023. All patients who received LTOWB during the 12-month study period were included. Patients initially excluded from receiving LTOWB per protocol included those under the age of 16, pulseless patients, those undergoing thoracotomy or receiving cardiopulmonary resuscitation (CPR), and women 50 years of age or younger. Each case of LTOWB administration was identified and reviewed, including the number of all blood components transfused (including LTOWB). In-hospital mortality was the primary outcome. Secondary outcomes included the total amount of blood components used and markers of coagulation. Data were collected from both a trauma registry and a blood bank registry. Duplicate and missing data were assessed by two independent reviewers. Study data were stored in accordance with institutional requirements and analyzed using appropriate statistics.

Results

From the initiation of the LTOWB program in December 2022 through the end of December 2023, 81 adults received LTOWB in the emergency department (ED) at our center, with an overall in-hospital mortality rate of 23.5% (19). The majority of recipients were African American males, median age 33 (interquartile range (IQR) age 24-45), with penetrating trauma. The median Injury Severity Score (ISS) on presentation was 17 (IQR 10-26). These patients received a median of two units of LTOWB (IQR 1-4 units) and a median of 4 total units of blood components (inclusive of the initial LTOWB; IQR 2-12 units) in the first 24 hours. No life-threatening transfusion reactions were observed.

Conclusions

During the first 12 months following implementation of an LTOWB protocol for trauma patients, 81 patients received whole blood during their initial resuscitation period for traumatic hemorrhage, with no cases of significant transfusion reactions observed within 24 hours following LTOWB administration, demonstrating LTOWB administration to be safe and feasible in trauma resuscitation. While survival bias may have skewed the impact of LTOWB implementation, assessment for improved outcomes in specific sub-groups within this cohort of patients will be better elucidated in future studies through comparative analysis.

## Introduction

Despite many advances, trauma remains one of the leading causes of death, accounting for approximately 8% of total deaths globally each year [1]. In the United States, unintentional injuries are the third leading cause of death across all age groups, totaling over 227,039 deaths in 2023 [2]. Hemorrhagic shock is one of the leading causes of mortality amongst trauma patients [3], and the majority of potentially preventable deaths from trauma are related to hemorrhage [4]. Our urban academic level 1 trauma center sees a high volume of trauma patients, a large proportion which is penetrating trauma, and many of whom are at risk of massive hemorrhage. Thus, finding additional means of minimizing harm from traumatic hemorrhagic shock is of high value.

Timely recognition of injuries and prompt management of hemorrhage through bleeding control and hemostatic resuscitation have a major impact on survival [5]. Based on evidence from military populations, low-titer group O whole blood (LTOWB) is making a resurgence for resuscitation of trauma patients with hemorrhage in civilian trauma centers because early data demonstrate potential mortality and coagulation benefit of LTOWB compared to balanced component transfusions [6-9]. Furthermore, early administration of LTOWB as an adjunct to component therapy to patients undergoing massive transfusion has demonstrated additional reduction in mortality [10-13].

While primarily retrospective and observational evidence supporting LTOWB is promising, the majority of prior studies in LTOWB usage have been in the military setting, and there remains limited evidence for its use in civilian trauma. These limitations include different patient populations, different injury patterns, and differences in types of whole blood products available, with military studies using primarily fresh/warm blood while civilian studies primarily using cold-stored whole blood. Continued study of both the utility and safety of LTOWB usage in civilian trauma centers is ongoing. We sought to contribute to the growing body of evidence surrounding LTOWB usage in civilian trauma by describing the natural experience of the first year of whole blood utilization at our urban, academic, civilian level 1 trauma center. Therefore, the primary objective of this study was to describe the patient population, injury patterns, and mortality outcomes for the first year of our LTOWB program. Secondary objectives were to quantify LTOWB and total blood product utilization, assess the feasibility and safety of our protocol, and evaluate for any acute transfusion reactions. 

## Materials and methods

Setting

This study is a single-site retrospective descriptive analysis of patients at an urban, academic-level 1 trauma center, over 12 months, from the start of program implementation to one year post-implementation. Our level 1 trauma center sees over 2,800 trauma patients annually [14]. The blood bank at our center experiences high utilization in part due to a combination of large volumes of trauma cases with massive transfusion protocol (MTP) activation, in addition to scheduled operating room (OR) cases with expected high-volume blood loss. A LTOWB protocol was developed in a multidisciplinary fashion between the departments of Surgery, Anesthesia, and Critical Care Medicine (ACCM), Emergency Medicine, and Pathology.

The protocol was initially intended for adult male trauma patients with a high risk of decompensation due to massive hemorrhage. Eligibility criteria included adult trauma patients presenting to the Emergency Department with either an Assessment of Blood Consumption (ABC) Score ≥ 2 or patients triggering MTP activation at the discretion of the trauma team leader. MTP at our institution constitutes as 6 units of red blood cells (RBC), 6 units of thawed plasma, and 1 unit of apheresis platelets per round of MTP. Patients were excluded if they were pulseless, receiving ED thoracotomy, receiving ongoing cardiopulmonary resuscitation (CPR), or were females of childbearing potential (FCP) as defined as less than or equal to 50 years old. Exclusion of women ≤50 years old was due to alloimmunization risk based on data available at the time of protocol creation. Additionally, pediatric patients were excluded by the local trauma activation system (adult and pediatric trauma patients are triaged to different areas of the hospital) rather than explicitly as part of the original protocol.

The LTOWB units were RhD positive, leukocyte-reduced using a platelet-sparing filter, had anti-A and anti-B antibody titers <200, and were used within 14 days of donation. A maximum of four units of LTOWB could be administered in the initial phase of resuscitation. Per protocol, it was expected that the MTP should be initiated simultaneously for patients who require multiple units of LTOWB. Up to eight units per week of LTOWB were pre-staged in blood refrigerators (HaemoBanks) adjacent to the trauma bay [15].

Study design and data collection

All trauma patients treated at our hospital are recorded under a state-wide trauma registry. Blood product administration is tracked in the trauma registry, electronic medical record (EMR), and blood bank laboratory information system. We conducted a retrospective review of trauma patients within the trauma registry, identifying all trauma patients who presented to the Johns Hopkins Hospital Emergency Department and received LTOWB from December 23, 2022, through December 31, 2023. The 12-month period corresponds to a one-year review starting from the initiation of the LTOWB program and covering the subsequent 12 months. Duplicate data and missing data were analyzed and resolved by the study team using two independent reviewers. Database use and statistical analysis were through a protected and de-identified trauma registry and blood bank database on the institutional Secure Analytic Framework Environment (SAFE) desktop.

The patient data were collected under a pre-existing IRB #00003794, parent ID NA_00078426, for the Johns Hopkins Hospital. Each instance of LTOWB administration was identified and studied, including the number of pre- and post-LTOWB units of blood components. Data collection and analysis were in compliance with institutional and international ethical standards, data anonymization, and patient confidentiality.

Statistical analysis

Following data collection, descriptive and summary analyses were performed with the assistance of a biostatistician and institutional SAS 9.4 software. Basic descriptive analysis performed, with mean ± standard deviation (SD) for normally distributed variables and median (interquartile range (IQR)) for non-parametric data. The main variables assessed were demographics of trauma patients that ultimately received LTOWB, including age, race, mechanism of injury, and presenting vital signs. Additional variables tracked included Injury Severity Score (ISS), disposition, survival to discharge, and survival at 24 hours. The number of blood products received in total, activation of MTP, initial ionized calcium level, and transfusion reactions were also accounted for. Thromboelastography (TEG) data were available for only a subset of patients, as the utilization of this test is not standardized for all trauma admissions. The power (n) for this subset of data is noted in the resulting data, and its limitations in applicability are noted.

## Results

From December 2022 through December 2023, a total of 81 trauma patients received LTOWB and were included in this study. The median age was 33 years (IQR 24-45), and patients were predominantly African American (64, 79%). Most of these patients (55, 67.9%) presented with penetrating trauma and went either to the operating room (30, 37.0%) or ICU (24, 29.6%) after leaving the emergency department (Table 1). The median ISS on presentation was 17 (IQR 10-26). In total, 19 (23.5%) patients died, with 10 (12.4%) dying in the emergency department. Overall survival rate to hospital discharge was 76.5%; however, it should be noted that in the study population, no patients who arrived in cardiac arrest survived to hospital discharge. When excluding those who arrived in cardiac arrest, the survival rate was 92.5%. The initial median SBP was 82 mmHg (IQR 54-111), the median shock index (SI) was 1.2 (IQR 0.9-1.4), and the median Glasgow Coma Scale (GCS) was 15 (IQR 7.5-15).

**Table 1:** Characteristics of recipients of whole blood (total n = 81). ED, emergency department; OR, operating room; ICU, intensive care unit; ICA, intermediate care area; GCS, Glasgow Coma Scale; LTOWB, low-titer O whole blood; SBP, systolic blood pressure; SI, Shock Index; ISS, Injury Severity Score; IQR, inter-quartile range; n, number

Gender	*n* (% of total)
Male	78 (96.3%)
Female	3 (3.7%)
Age (years)	
Male, range	13-73
Female, range	75-91
Overall, median (IQR)	33 (24,45)
Race	*n* (% of total)
African American/Black	64 (79.0%)
White	9 (11.1%)
Other	8 (9.9%)
ED Disposition	*n* (% of total)
OR	30 (37.0%)
ICU	24 (29.6%)
Floor	10 (12.4%)
Morgue/Died	10 (12.4%)
ICA, Telemetry, Step-Down	4 (4.9%)
Observation	2 (2.5%)
Transferred	1 (1.2%)
Discharge Disposition	*n* (% of total)
Home or Self-Care	23 (28.4%)
Medical Examiner/Morgue	19 (23.5%)
Home with Services	13 (16.1%)
Skilled Nursing Facility	13 (16.1%)
Inpatient Rehab Facility	5 (6.2%)
Specialty Referral Center	3 (3.7%)
Jail	2 (2.5%)
Against Medical Advice	2 (2.5%)
Psychiatric Hospital or Psychiatric Unit	1 (1.2%)
Survival	*n* (% of total)
Survival to Hospital Discharge	62 (76.5%)
Survival to Discharge, excluding prehospital arrests	62 (92.5%)
Survival to Discharge for prehospital arrests	0 (0%)
Survival to Discharge for LTOWB only	34 (94.4%)
Location of Termination of Resuscitation	*n* (% of total)
ED	10 (52.6%)
OR	4 (21.1%)
ICU	5 (26.3%)
Injury Type	*n* (% of total)
Penetrating	55 (67.9%)
Blunt	26 (32.1%)
Patient GCS	
GCS on Arrival, median (IQR)	15 (7.5,15)
GCS 3 on Arrival, *n* (%)	19 (23.5%)
GCS 15 on Arrival, *n* (%)	40 (49.4%)
Patient Characteristics on Arrival	
Arrived in Cardiac Arrest, *n* (%)	14 (17.3%)
SBP on Arrival (mmHg), median (IQR) (*n* = 80)	82 (54, 111)
SI on Arrival, median (IQR) (*n *= 65)	1.2 (0.9, 1.4)
ISS, median (IQR)	17 (10, 26)

**Figure 1:**
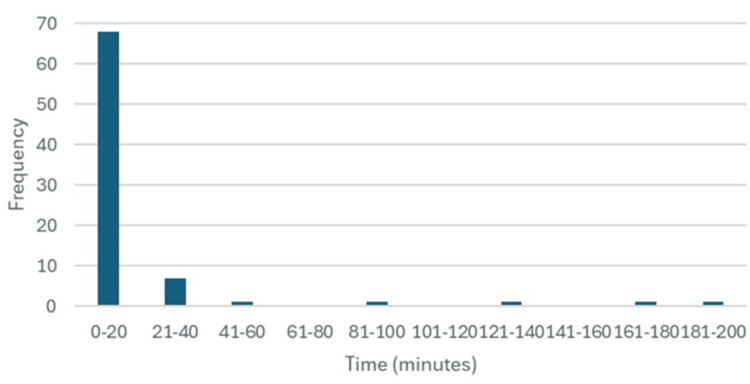
Time to WB administration. WB, whole blood

The median use of LTOWB was two units (IQR 1-4 units) per patient, with a median time from arrival to transfusion of the first unit of LTOWB of 5.5 minutes (IQR 2.00-12.25 minutes) (Table 2). Notably, while the majority of patients received LTOWB within the first 20 minutes of arrival (68, 85%), some patients did not develop hemorrhagic shock until later in their stay (Figure 1). Four patients (5%) received their first unit of whole blood after more than one hour from arrival time. Overall, patients were able to receive LTOWB early into the presentation to our ED.

**Table 2:** Transfusion data of recipients of WB. WB, whole blood; MTP, massive transfusion protocol; ultra-massive transfusion, >100 units of total blood products; IQR, interquartile range; min, minutes; n, number

	Median (IQR)	Total number of pts
Time from Door (Arrival) to first WB Unit Administration	5.5 minutes (2.00, 12.25)	*n* = 80
Time from Door to MTP Initiation	6.00 minutes (2.50, 7.00)	*n* = 15
24 Hour Total Blood Product Usage	4 units (2, 12)	*n* = 81
24 Hour WB Usage	2 units (1, 4)	*n* = 81
Total Blood Product Usage	5 units (2, 13)	*n* = 81
Total WB Usage	2 units (1, 4)	*n* = 81
	*n* (% of total)	Total number of pts
Number of total MTP Activation	45 (56%)	*n* = 81
Ultra-massive Transfusion	2 (2.50%)	*n* = 81
Transfusion Reaction During Admission	2 (2.50%)	*n* = 81

The median use of all blood products (including RBC, FFP, platelets, and cryoprecipitate) was four units (IQR 2-11 units) per patient within 24 hours and five units (IQR 2-13 units) within 72 hours (Table 2). Comparing survivors to non-survivors, on average, the survivors received an LTOWB to component ratio of 1:4, whereas non-survivors had a ratio of 1:3. Of the 81 patients who received LTOWB, 41 (51%) went on to receive MTP, with a median time from door to MTP activation of six minutes (IQR 2.5-7 minutes). Of note, two of the 81 patients in this cohort underwent ultra-massive transfusion (>100 units of any blood component). These ultra-MTP patients each received over 175 units of blood components. They both remained alive after six hours but died within 24 hours.

TEG data were available for approximately half of the patients (Table 3). While some patients had severe derangements, median values were mostly within normal limits, except for R time (median 3.85 minutes, IQR (3.25, 4.80); ref: 4.0-9.0 minutes). The total number of patients with available data for each parameter is noted in Table 3 under the *total *column.

**Table 3:** TEG parameters and ionized calcium levels of recipients of whole blood. TEG, thromboelastography; iCal, ionized calcium; IQR, interquartile range

Ionized Calcium (iCal) levels	Median (IQR)	Total	Reference value
iCal (mmol/L)	1.07 (0.97,1.17)	*n* = 60	1.13-1.32 mmol/L
TEG parameters			
Clot Form (R) Time (minutes)	3.85 (3.25, 4.80)	*n* = 42	4.0-9.0 minutes
Clot Firm (K) Time (minutes)	1.70 (1.30, 2.40)	*n* = 41	1.0-3.0 minutes
Clot Strength (Alpha Angle) (deg)	68.15 (59.48, 71.80)	*n* = 42	57.0-76.0 degrees
Clot Strength [Max Amplitude) (mm)	60.20 (49.90, 62.20)	*n* = 42	52.0-75.0 mm
Clot Lysis at 30 minutes	0.05% (0.00, 0.65)	*n* = 42	0-10%
Coagulation Index	1.10 (-0.70, 2.50)	*n* = 37	-3 to 4

Three LTOWB recipients were female, with ABO/Rh types of O+, O+, and A+. All three were older than 50 years, and all three suffered blunt trauma. All of the patients in this study were followed by the blood bank throughout their hospitalization for potential transfusion reactions. Two of the 81 patients who received LTOWB in our study had transfusion reactions during their admission; however, none had a temporal association with their whole blood transfusions. One patient had a febrile non-hemolytic transfusion reaction to plasma while being administered on hospital day five. The other sustained transfusion-associated circulatory overload after transfusion of nine units of platelets on hospital day two.

Overall, results demonstrate that LTOWB administration was feasible, rapidly available for early transfusion within the 1st 20 minutes, and overall safe with no significant transfusion reactions in first 24 hours. 

## Discussion

Our initial experience demonstrates that the implementation of an LTOWB protocol in a civilian level 1 trauma center is feasible, safe, and rapidly deliverable at the initial point of resuscitation. The resuscitation of victims of hemorrhagic shock has transitioned over the last century from whole blood transfusion to component therapy. However, recently, there has been a renewed focus on cold-stored low-titer anti-A and anti-B O+ whole blood. Within the last decade, whole-blood availability has significantly expanded in trauma centers and the prehospital arena. In a 2021 survey of level 1 trauma centers, of those who responded, 42% were currently utilizing LTOWB, and another 14% were planning on implementing it soon [16]. In Maryland, State Police medevac helicopters and several county-based emergency medical services (EMS) agencies have recently started carrying whole blood to resuscitate patients in hemorrhagic shock, though, of note, none of the patients in our study received any pre-hospital blood products. These services are among the over 240 EMS services in the country that currently carry blood products [17]. 

Mortality in trauma patients from severe hemorrhage remains high, up to 50% of associated deaths. Early resuscitation with a balanced ratio of blood products is critical and is associated with decreased mortality [12]. At our center, whole blood was able to be administered early in a patient’s resuscitation at a median time of 5.5 minutes from time of arrival to administration. In one study of the American College of Surgeons Trauma Quality Improvement Program databank, examining over 1,000 victims of trauma in shock who received whole blood and MTP, earlier time to first whole blood transfusion was associated with reduced mortality at 24 hours and 30 days [12]. Torres et al. found that the largest drop in survival was seen when whole blood was transfused more than 14 minutes from the time of arrival. In their study, the median time to first whole blood transfusion was 30 minutes longer than in our cohort. Our early LTOWB administration can be attributed to the proximity of LTOWB-containing remote blood refrigerators to our trauma bays and our focus on blood-first resuscitation for hemorrhagic shock. Our study demonstrated that early administration of LTOWB is feasible in a civilian trauma center and could raise potential implementation in standard trauma protocols in other centers, although there may be several logistical barriers in broader implementation, including LTOWB availability and storage capacity of cold-stored LTOWB in their respective trauma bay. 

In the cohort of patients given LTOWB at our center, the majority were young African American men with penetrating trauma. The majority arrived in shock and with severe injuries. Gun violence is a pandemic plaguing the entire United States and Baltimore specifically, with over 203 gun-related homicides in Baltimore City in 2023 [14,18]. Injuries from firearms are associated with higher mortality and blood product usage compared to other mechanisms, underscoring the importance of access to early balanced blood resuscitation at our center and others with similar proportions of penetrating trauma [19].

While the overall survival rate of the patient cohort studied was 76.5%, if excluding those who arrived in cardiac arrest, the survival rate was 92.5%. This is despite an overall severely injured patient population, with an initial median SBP of 82 mmHg, a median SI of 1.2, and a median ISS of 17. As the focus of this study was to simply describe the population of trauma patients who received LTOWB upon arrival during the first 12 months of protocol implementation at our center, there is no direct comparator or control in this population. This limits the ability of this study to draw an association regarding any added benefit or advantage of whole blood over component therapy in this population of trauma patients. However, there is a plan for a future propensity-matched study of this same population, to include a control group of patients of similar demographics and severity of injury that presented to our center during the study period and would have qualified for LTOWB per protocol but only received component blood products. Such a study will yield greater ability to identify the benefit, if any, of LTOWB over component therapy. 

There were protocol deviations, notably in the case of traumatic cardiac arrest. Our protocol initially excluded patients who arrived in cardiac arrest to try to make the most efficacious use of a limited LTOWB supply, but 14 (17%) of the 81 patients who received LTOWB arrived initially pulseless. Aligned with data showing poor prognosis for victims of traumatic cardiac arrest, none of these patients survived [20]. However, Torres et al. demonstrated there may be a benefit of early LTOWB in some patients suffering from traumatic cardiac arrest [12]. As the availability of LTOWB increases, there may be opportunities to broaden this protocol’s inclusion criteria. With increased utilization, there will be a greater amount of data from which to draw important information regarding LTOWB usage in trauma, such as identification of which type of trauma patient population yields the greatest benefit from LTOWB over component therapy. This will allow maximal efficacy of this limited but potentially powerful resource. 

Limitations

This study has several limitations. This is a retrospective descriptive analysis with inherent flaws related to data precision and availability. Specifically, we had some missing data for door-to-LTOWB time and TEG values, given that we do not collect TEGs by default on trauma patients. As with most single-center studies, there are limitations in generalizability, especially when comparing to centers with lower volumes of penetrating trauma. There are further limitations regarding the study population that might limit generalizability: predominantly young African American males and penetrating mechanisms of injury. Additionally, at the time of protocol creation, females of childbearing age (typically defined as under 50 years of age) were excluded due to concerns about D-alloimmunization and the associated small risk of hemolytic disease of the fetus and newborn (HDFN) in future pregnancies among RhD-negative individuals. Thereby, a large percentage of the adult population could have potentially been excluded as a result, although at the time of protocol implementation, this was the overall practice of most civilian centers using LTOWB [21]. Finally, when studying patients in hemorrhagic shock, a significant limiting factor is survival bias. Patients who had markedly high ISS and SI on arrival, with a low chance of survival, were unlikely to benefit from any intervention. Included in this is the skewing effect of patients who went on to receive ultra-massive transfusion during their first 24 hours of presentation.

## Conclusions

In this observational, descriptive analysis of LTOWB use among adult trauma patients during the first 12 months following implementation of a whole blood protocol at our academic Level I trauma center, prompt access to blood allowed near-immediate administration, and, excluding patients who experienced traumatic cardiac arrest, overall survival was 92%. No patients in the study had evidence of transfusion reaction or transfusion-related complication within 24 hours of receiving LTOWB. Overall, this study showed that an LTOWB protocol can be effectively and safely implemented in a civilian trauma center, with rapid delivery of LTOWB at the time of arrival, and could demonstrate the feasibility and potential application of similar LTOWB protocols in other civilian trauma centers. Further investigation of the cohort using propensity-matching will identify any potential advantages of LTOWB over component therapy in terms of overall blood product usage, mortality, and other endpoints indicative of appropriate trauma hemostatic resuscitation. With a broader perspective, multi-center studies, this work could help inform hospital transfusion policies, trauma resuscitation algorithms, and regional trauma system preparedness regarding the appropriate use of LTOWB.
